# Modulating cardiac physiology in engineered heart tissue with the bidirectional optogenetic tool BiPOLES

**DOI:** 10.1007/s00424-023-02869-x

**Published:** 2023-10-21

**Authors:** Barbora Schwarzová, Tim Stüdemann, Muhammed Sönmez, Judith Rössinger, Bangfen Pan, Thomas Eschenhagen, Justus Stenzig, J. Simon Wiegert, Torsten Christ, Florian Weinberger

**Affiliations:** 1 Department of Experimental Pharmacology and Toxicology, University Medical Center Hamburg-Eppendorf, Martinistr. 52, 20246 Hamburg, Germany; 2 German Centre for Cardiovascular Research (DZHK), Partner Site Hamburg/Kiel/Lübeck, Berlin, Germany; 3 Research Group Synaptic Wiring and Information Processing, Center for Molecular Neurobiology Hamburg, University Medical Center Hamburg-Eppendorf, Hamburg, Germany; 4 Department of Neurophysiology, Mannheim Center for Translational Neuroscience, Medical Faculty Mannheim, Heidelberg University, Mannheim, Germany

**Keywords:** Optogenetic, Cardiac physiology, Tissue engineering, Stem cells

## Abstract

Optogenetic actuators are rapidly advancing tools used to control physiology in excitable cells, such as neurons and cardiomyocytes. In neuroscience, these tools have been used to either excite or inhibit neuronal activity. Cell type–targeted actuators have allowed to study the function of distinct cell populations. Whereas the first described cation channelrhodopsins allowed to excite specific neuronal cell populations, anion channelrhodopsins were used to inhibit neuronal activity. To allow for simultaneous excitation and inhibition, opsin combinations with low spectral overlap were introduced. BiPOLES (Bidirectional Pair of Opsins for Light-induced Excitation and Silencing) is a bidirectional optogenetic tool consisting of the anion channel Guillardia theta anion-conducting channelrhodopsin 2 (GtACR2 with a blue excitation spectrum and the red-shifted cation channel Chrimson. Here, we studied the effects of BiPOLES activation in cardiomyocytes. For this, we knocked in BiPOLES into the adeno-associated virus integration site 1 (AAVS1) locus of human-induced pluripotent stem cells (hiPSC), subjected these to cardiac differentiation, and generated BiPOLES expressing engineered heart tissue (EHT) for physiological characterization. Continuous light application activating either GtACR2 or Chrimson resulted in cardiomyocyte depolarization and thus stopped EHT contractility. In contrast, short light pulses, with red as well as with blue light, triggered action potentials (AP) up to a rate of 240 bpm. In summary, we demonstrate that cation, as well as anion channelrhodopsins, can be used to activate stem cell–derived cardiomyocytes with pulsed photostimulation but also to silence cardiac contractility with prolonged photostimulation.

**Supplementary Information:**

The online version contains supplementary material available at 10.1007/s00424-023-02869-x.

## Introduction

Classically, modulation of cardiomyocyte physiology was achieved pharmacologically [10, 17, 34]. Many drugs have a rather complex mode of action, making interpretation sometimes challenging [26]. Optogenetics represents a newer modality to control excitable cells [32, 33, 54]. Optogenetic actuators are light-sensitive proteins that can be used to transiently perturbate electrical activity and thereby control cardiomyocyte physiology [1, 7, 12]. These novel tools can overcome constraints of pharmacological strategies as they allow to reversibly activate and inhibit cardiomyocytes within milliseconds (high temporal resolution) but also enable to distinctly target cell subpopulations (high spatial resolution). The aim of this study was to investigate the physiological properties of the optogenetic tool BiPOLES (Bidirectional Pair of Opsins for Light-induced Excitation and Silencing) on induced pluripotent stem cell–derived cardiomyocytes [47]. BiPOLES is a bidirectional optogenetic tool consisting of the anion channelrhodopsin GtACR2 (excitation maximum 470 nm) [16] and Chrimson, a red-shifted cation channelrhodopsin (excitation maximum 590 nm) [23], that are linked via the transmembrane β helix of the rat gastric H^+^/K^+^ ATPase and a mCerulean3 fluorescent label. Membrane trafficking is enhanced via a canonical Kir2.1 trafficking sequence (TS) in the linker region [2, 47]. BiPOLES has been used to demonstrate the applicability of bidirectional switchable bicolor emission organic light-emitting diodes [8] and ultrasonically controlled microsystems [41] but also to establish an all-optical voltage clamp that allowed to control muscular and neuronal activity [2] but has not been used in cardiomyocytes yet.

Light-gated cation channels, like channelrhodopsin2 [32] and its red-shifted derivatives, have successfully been applied to excite cardiomyocytes in various models [1, 7, 22, 35, 37, 48, 51], ranging from pluripotent stem cell–derived cardiomyocytes, engineered cardiac constructs [11, 27, 37], to rodents [7, 35, 48] and zebrafish [1]. Applications included toxicity screening [38], disease modelling [27, 37], and the development of optogenetic defibrillation strategies [6, 36]. Optogenetic anion channels have been identified and developed later [3, 16, 49, 50]. In neuroscience, the anion channelrhodopsins GtACR1 and GtACR2 (Guillardia theta anion channelrhodopsin1 and 2), have been applied to silence electrical activity in a variety of neuron populations by Cl^−^-driven photocurrents [2, 16, 30]. In neonatal rat cardiomyocytes, GtACR1 and GtACR2 activation inhibited contractility [15]. Similarly, prolonged GtACR1 activation inhibited contractility in ventricular rabbit cardiomyocytes. This stop in contractility resulted from a depolarization block [24]. In accordance, pulsed photostimulation could be used to evoke AP and pace isolated cardiomyocytes and zebrafish hearts. However, anion channelrhodopsins have not been applied as widely as their cation-conducting counterpart and have not been studied in stem cell–derived cardiomyocytes so far. This is of particular interest because the data from rabbit myocytes and zebrafish hearts indicates that anion channels might constitute a powerful tool to optogenetically control beating frequency for disease modelling (e.g., atrial fibrillation) as well as for drug toxicity screens in in vitro models. Here, we show that prolonged photostimulation of BiPOLES EHTs with either blue or red light caused a depolarization block and inhibited contractility, while pulsed photostimulation optically paced BiPOLES EHTs up to a frequency of 270 bpm. Thus, we show that both anion and cation currents could drive depolarization of cardiomyocytes and contraction in EHTs, while prolonged photostimulation resulted in a depolarization block and inhibition of EHT contractility.

## Methods

### Human iPSC culture and cardiac differentiation

UKEi001-A human iPSC line was reprogrammed with a Sendai virus–based method (CytoTune iPS Sendai Reprogramming Kit, Thermo Fisher). Human iPSCs were expanded in FTDA medium on Geltrex-coated cell culture vessels (Gibco, A14133-02) [13]. For the current study, a 3D embryoid body–based or a 2D monolayer cardiac differentiation protocol was used [4, 31]. For the 3D protocol, formation of embryoid bodies was performed in spinner flasks, followed by differentiation in Pluronic F-127–coated (Sigma-Aldrich, P2443) cell culture vessels with a sequential administration of growth factor– and small molecule–based cocktails to induce mesodermal progenitors, cardiac progenitors, and cardiomyocytes. For the monolayer differentiation protocol, iPSC cultured in FTDA were overlayed with Matrigel once they reached 60–70% confluency. This was followed by a sequential application of growth factors and small molecules to initially activate and subsequently inhibit the WNT pathway. Dissociation of differentiated cardiomyocytes was performed with collagenase.

### Generation of the BiPOLES-iPSC-line

Transgene integration in the AAVS1 locus was performed with CRISPR/Cas9 as recently described [44]. BiPOLES donor plasmid was provided by S.W. AAVS1-CAG-hrGFP was a gift from Su-Chun Zhang (Addgene plasmid #52344). BiPOLES was cloned into the AAVS1-CAG-hrGFP plasmid for CRISPR/Cas9 editing using the In-Fusion HD cloning kit (Takahara Bio). The BiPOLES plasmid then contained GtACR2, Chrimson, mCerulean3, the Kir2.1 membrane trafficking signal, the transmembrane β helix of the rat gastric H^+^/K^+^ ATPase under the control of a CAG promoter, homology arms (~800 bp in size each) for the AAVS1 locus, and a puromycin resistance cassette upstream of the promoter [39]. A single guide RNA (sgRNA) targeted the AAVS1 locus between exon 1 and 2 (sequence: GTCACCAATCCTGTCCCTAG). Nucleofection with the Cas9 ribonucleoprotein (IDT 1081059) was conducted using a 4D-Nucleofector (Lonza) according to the manufacturer’s protocol. Positively edited cells were either enriched by puromycin selection (InvivoGen, QLL-38-04B) or via FACS, based on mCerulean3 fluorescence. iPSCs were subsequently clonally expanded in mTeSR+ supplemented with Y-27632 (Biorbyt, orb154626) and master and working cell banks were generated from two clones. One clone was used for this study.

### Genotyping

PCR (Bioline, BIO-21106) amplification followed by Sanger sequencing (Eurofins Genomics) was used to verify correct transgene integration. Nanostring (Nanostring nCounter Sprint) analysis was performed for karyotyping.

### Flow cytometry

Single-cell suspensions of iPSCs or iPSC-derived cardiomyocytes were used for flow cytometry. iPSCs were blocked with 5% FBS (Biochrom, S0615) in PBS and stained. Cardiomyocytes were fixed in Roti®-Histofix 4% (Roth, P087) for 20 min at 4 °C and transferred to FACS buffer, containing 5% FBS, 0.5% saponin (Sigma-Aldrich, 47036), and 0.05% sodium azide (Sigma-Aldrich, 71290) in PBS. Samples were analyzed with a BD FACSCanto™ II Flow Cytometer and the BD FACSDiva™ Software 6.0 or BD FlowJo™ V10. In brief, cells were selected by gating SSC-A vs. FSC-A. Then, single cells were gated with FSC-H vs. FCS-A, before gating for fluorescence intensity using isotype-stained cells as control.

### Engineered heart tissue generation and analysis

EHTs were generated from cells and fibrinogen/thrombin (Sigma-Aldrich, F8630 and 605157) as previously described [40]. UKEi001-A or BiPOLES cardiomyocytes were digested with collagenase II (Worthington, LS004176; 200 U/ml) in Ca^2+^-free HBSS (Gibco, 14175-053) with 1 mM HEPES (Sigma-Aldrich, 9105; pH 7.4), 10 μM Y-27632, and 30 μM *N*-benzyl-*p*-toluene sulfonamide (TCI, B3082)) for 3.5 h at 37 °C (5% CO_2_, 21% O_2_). The dissociated cells were resuspended in Ca^2+^-containing DMEM (Biochrom, F0415) with 1% penicillin/streptomycin (Gibco, 15140). Cell concentration was adjusted to 10–15×10^6^ cells/ml. Fibrin-based human EHTs were generated in agarose casting molds with solid silicone racks [42] (100 μl per EHT, 1 × 10^6^ cells). After ~7 days in culture, human EHTs displayed spontaneous coherent, regular beating deflecting the silicone posts that allowed video-optical contraction analysis. Force measurement and photostimulation of BiPOLES EHTs were performed as described by Lemme et al. [27]. In brief, analysis of contractile force was performed with a video-optical recording system. For baseline measurements, the contraction peak analysis was performed during spontaneous beating either under low intensity green (525 nm, irradiance 0.001 mW/mm^2^) or infrared (>750 nm) light illumination. Optical pacing was performed with an LED system custom made for the video-optical recording system. With this system, it was possible to stimulate with either blue (470 nm) or red (635 nm) light. Maximal irradiance was 0.013 mW/mm^2^ for blue and 0.010 mW/mm^2^ for red light. Stimulation was performed with maximal light intensity. Electrical pacing was performed with custom-made pacing electrodes for 24 well plates. All-trans-retinal (Sigma-Aldrich, R2500) was supplemented for some experiments. Continuous optical stimulation was performed with the custom-made LED system or with the illumination system pe-4000 (CoolLED).

### Histology

Cells were fixed with Roti®-Histofix 4% for 10 min followed by blocking with 3% milk powder (Sigma-Aldrich, 70166) in TBS (Sigma-Aldrich, T6664) and 0.1% Triton X-100 (Roth, 3051.3) and primary antibody incubation in the same buffer. After 24 h at 4 °C, cells were washed three times and a secondary antibody and 4′,6-diamidine-2′-phenylindole dihydrochloride (DAPI, Biochemica, A1001 0025) were applied. After 1 h at room temperature, cells were washed with PBS and imaged.

EHTs were fixed in Roti®-Histofix 4% for up to 24 h and subsequently embedded in 4% agarose (Thermo Fisher, 15510-027) for vibratome sectioning in 60 μm sections (Leica). Next, the sections were blocked with 5% BSA (Fisher Scientific, 30036578) in TBS followed by antibody incubation in the same solution for 24 h at 4 °C. Sections were washed three times with PBS. Secondary antibody and DAPI incubation were performed for 1 h at room temperature. Sections were washed three times with PBS and mounted on glass slides using Fluoromount-G (Invitrogen).

### Action potential measurements

Sharp microelectrodes were used to record AP as described previously [28] in EHTs superfused with Tyrode’s solution containing (in mmol/l) NaCl 127, KCl 5.4, MgCl_2_ 1.05, CaCl_2_ 1.8, Glucose 10, NaHCO_3_ 22, NaHPO_4_ 0.42, equilibrated with O_2_–CO_2_ (95:5) pH 7.4 at 36.5 ± 0.5 °C. The pipettes had a resistance of 20 to 50 MΩ when filled with 3 mol/l KCl. Signals were amplified by a BA-1s npi amplifier (npi electronic). Lab-Chart software (ADInstruments) was used to record and analyze AP. At least five consecutive AP were averaged for calculation of AP parameters. AP recordings were performed without background illumination.

### Statistics

Statistical analyses were performed with Prism 9 (GraphPad software). Comparison among two groups was made by two-tailed unpaired Student’s *t* test. One-way ANOVA followed by Tukey’s test for multiple comparisons was used for more than 2 groups. Error bars indicate SEM. *p*-values are displayed graphically as follows: *p < 0.05, **p < 0.01, ***p < 0.001.

## Results

### Generation of cardiomyocytes expressing BiPOLES

**Fig. 1:**
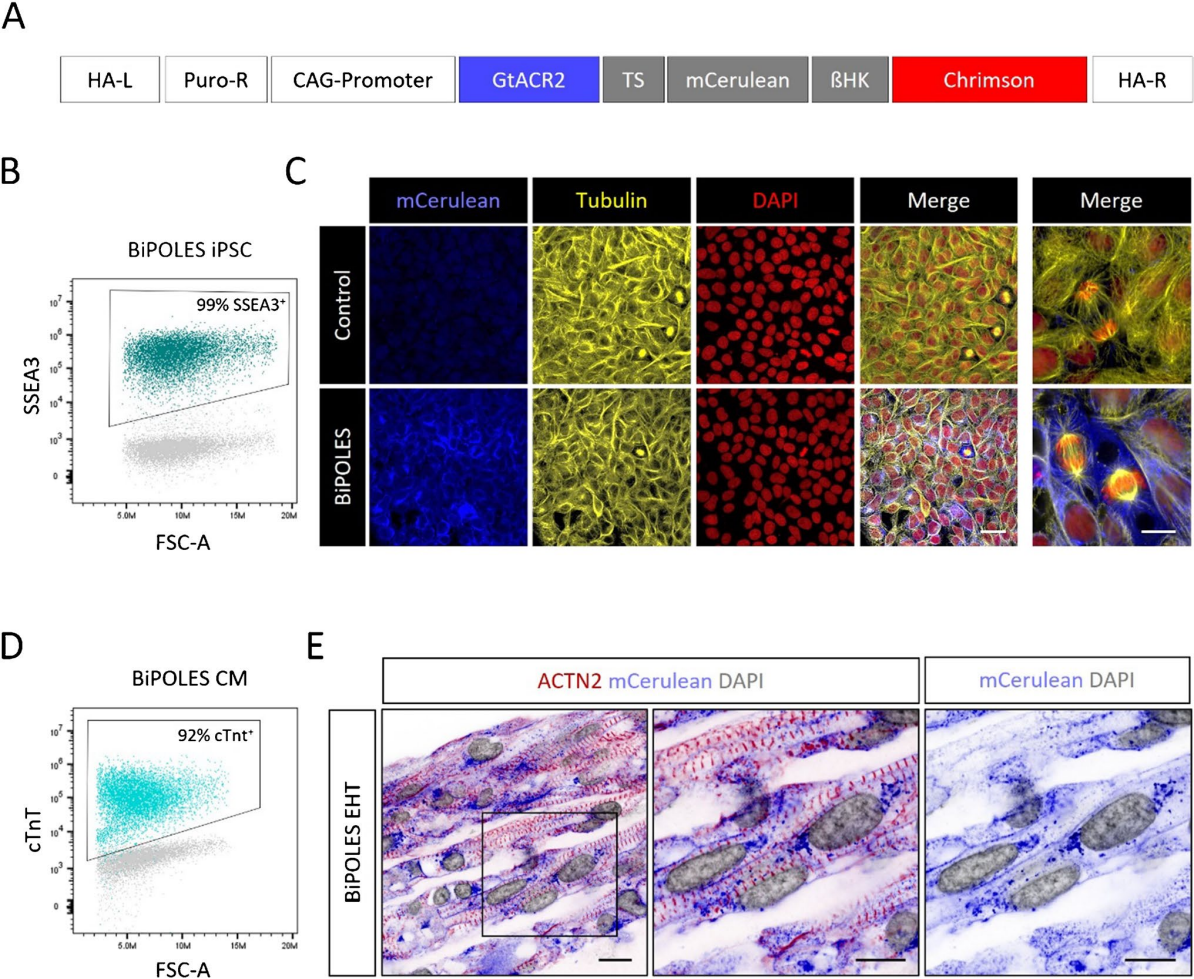
Generation of BiPOLES pluripotent stem cell–derived cardiomyocytes. **A** Scheme of the BiPOLES knock-in plasmid (HA-L, homology arm left; HA-R, homology arm right; Puro-R, puromycin resistance cassette; GtACR2, Guillardia theta anion channelrhodopsin2; TS, Kir2.1 trafficking sequence; ßHK, transmembrane β helix of the rat gastric H^+^/K^+^ ATPase). **B** Flow cytometry of BiPOLES iPSC stained for stage specific embryonic antigen 3 (SSEA3). Isotype control is shown in grey. **C** Immunohistology of control and BiPOLES hiPSC in lower (4 left images) and higher magnification (right image). **D** Flow cytometry of BiPOLES iPSC–derived cardiomyocytes stained for cardiac troponin T (cTnT). Isotype control is shown in grey. **E** Longitudinal section of an engineered heart tissue derived from BiPOLES-cardiomyocytes. The inset in the left image is shown in higher magnification on the right. ACTN2, α-actinin-2; DAPI, 4′,6-diamidino-2-phenylindol. Scale bars: 20 μM in **C** (low magnification) and 10 μM in **C** (high magnification) and **E**

We engineered induced pluripotent stem cells with CRISPR/Cas9 and knocked-in BiPOLES in the AAVS1 locus of human iPSC (iPSC line UKEi001-A, Fig. 1A). The BiPOLES iPSC line retained pluripotency (stage specific embryonic antigen 3 (SSEA3) positivity >99%, Fig. 1B) and uniformly expressed the transgene (Fig. 1C). Nanostring analysis revealed a regular karyotype (Supplemental Figure 1). BiPOLES iPSC could be differentiated to cardiomyocytes with high purity (cardiac troponin-t (cTnT) positivity 92% on average, Fig. 1D). Engineered heart tissue (EHT) was cast for functional characterization and to study the impact of channel activation. BiPOLES EHTs developed similar to control EHTs derived from unedited cardiomyocytes from the parental iPSC line. Cardiomyocytes matured, aligned along the force line, and developed regular sarcomeric structure. Histological analysis showed a preferentially membranous BiPOLES localization (Fig. 1E).

### Physiological characterization of BiPOLES EHTs

**Fig. 2:**
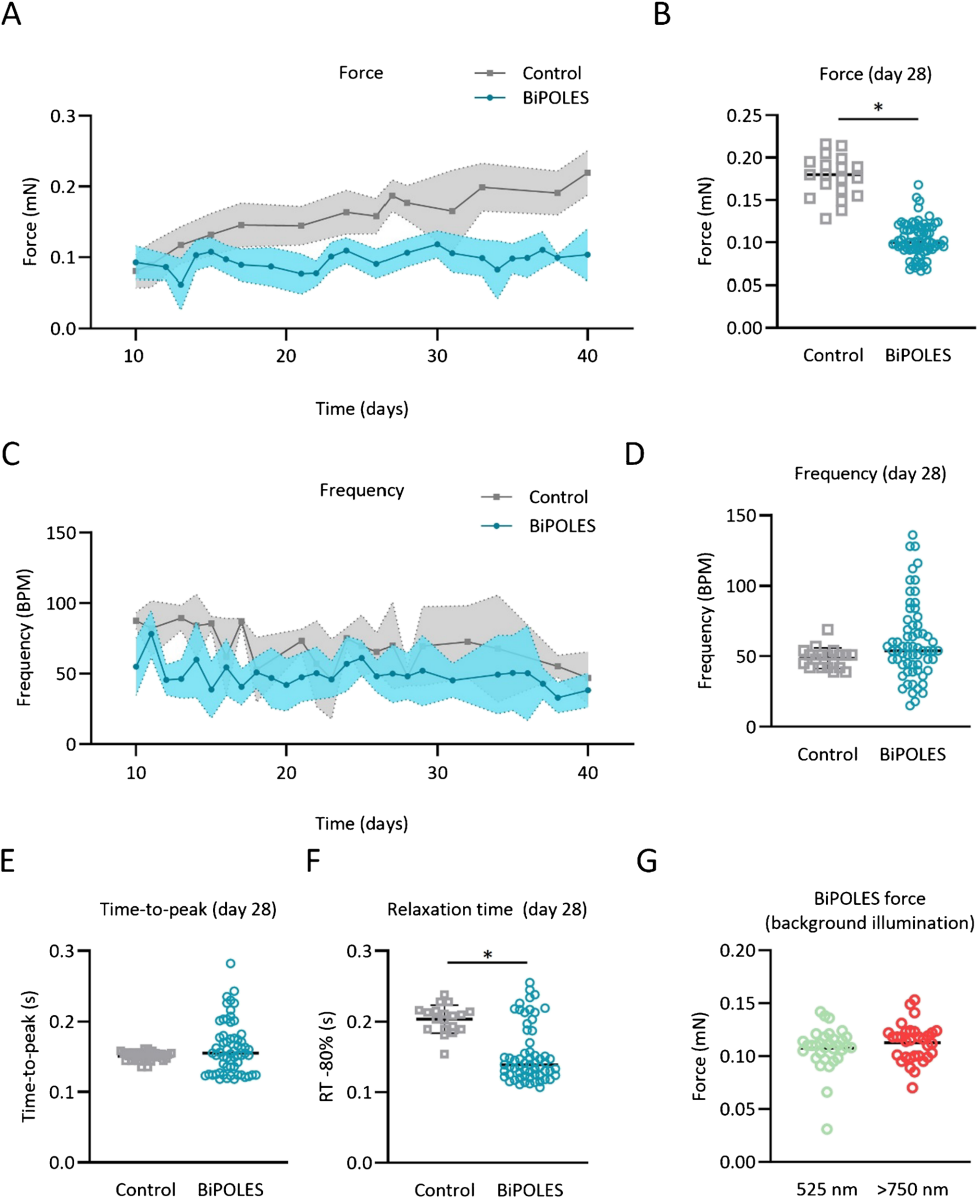
Physiological characterization of BiPOLES EHTs. **A** Contractility measurement of BiPOLES and control EHTs over time. **B** Comparison of contractility between BiPOLES EHTs and control EHTs after 1 month in culture. **C** Beating frequency of BiPOLES and control EHTs over the culture period. **D** Comparison of beating frequency between BiPOLES and control EHTs at day 28 of culture. **E** Comparison of contraction time (time-to-peak) and **F** relaxation time at day 28 of culture. **A**–**G** One EHT represents one data point. Depicted is data from three BiPOLES EHT batches (*n* = 14–16 EHTs per batch) and one unedited control batch (*n* = 19 EHTs). An unpaired two-sided Student’s *t* test was used for statistical analysis. Light intensity (525 nm) for background illumination was 0.001 mW/mm^2^

EHTs started to beat coherently after ~7 days in culture. Over the culture period of 40 days BiPOLES EHTs developed lower contractile force than control EHTs (Fig. 2A). Average force after 1 month in culture was 0.10 ± 0.02 mN in BiPOLES EHTs compared to 0.18 ± 0.02 in control EHTs (Fig. 2B). Frequency decreased over time, similar as in control EHTs (Fig. 2C). Beating frequency between individual BiPOLES EHTs showed a greater variability, but on average, the beating frequency did not significantly differ from control EHTs at day 28 in culture (49 ± 7 bpm in control EHTs vs. 61 ± 27 bpm in BiPOLES EHTs; Fig. 2D). A more detailed analysis of force recordings revealed that contraction time (time-to-peak), even though more variable in BiPOLES EHTs, did not differ from control EHTs, but relaxation time was shorter in BiPOLES EHTs (Fig. 2E and F). It should be noted that force measurements in EHTs were performed by video-optical recording (see “Methods”). To enable video recordings, minimal background illumination was needed. For this purpose, we used a low-intensity green backlight illumination (0.001 mW/mm^2^). To study whether the green backlight had any effect on BiPOLES EHTs, we compared measurements under low intensity green light illumination (0.001 mW/mm^2^) with measurements performed under an infrared backlight illumination (which was not possible with the regular set-up) for one BiPOLES EHT batch. There was no difference in contractile force, indicating that low-intensity green light had no substantial effect on cardiomyocyte contractility (Fig. 2G).

### Optical stimulation of BiPOLES EHTs

**Fig. 3:**
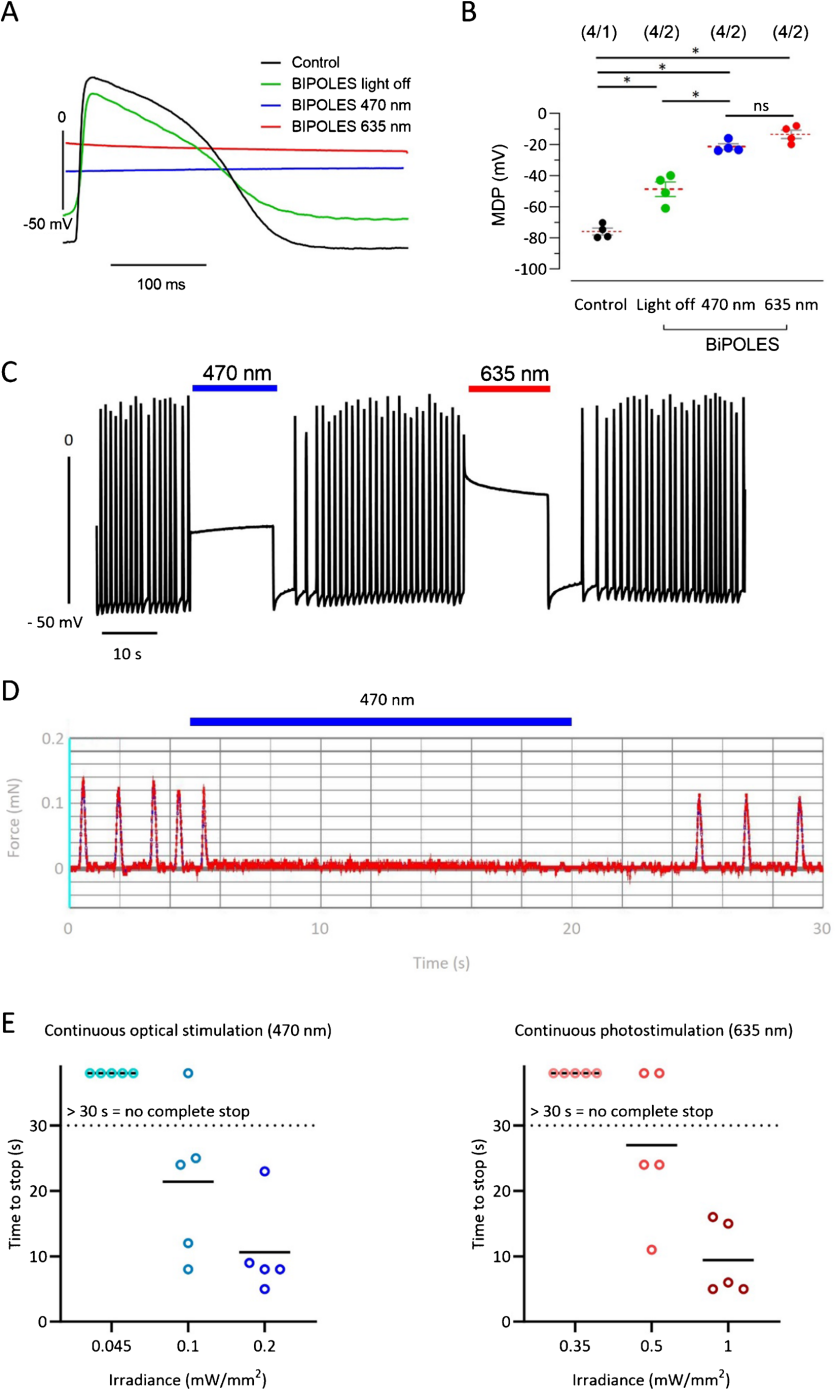
Effects of prolonged photostimulation on BiPOLES EHTs. **A** Example of membrane potential recordings in control and BiPOLES EHTs. **B** Maximum diastolic potentials (MDP) under prolonged photostimulation with blue light (470 nm) and red light (635 nm). Mean values ± SD (*n*/*n* indicates number of EHTs/number of batches). Irradiance 1–2 mW/mm^2^. **C** AP recorded in low speed in a BiPOLES EHT illuminated with blue (470 nm) and red light (635 nm) as indicated by bars. **D** Force recording of a BiPOLES EHT under continuous blue light illumination. Irradiance 0.013 mW/mm^2^. **E** Time to stop of EHT contractility under blue (470 nm) and red light (635 nm) with increasing irradiance. Photostimulation was performed for a maximum of 30 s per EHT

First, we explored whether expression of BiPOLES in EHTs influenced AP shape. In contrast to the contractility analysis, the AP measurement setup allowed to, once an AP recording was established, turn off light completely [28]. We then measured effects of continuous photostimulation with either blue (470 nm) or red light (635 nm) on AP characteristics. Of note, most experiments were performed with 635 nm which does not perfectly match the excitation maximum of Chrimson, and only during later experiments (e.g., when constructing a strength–duration curve of optical pacing threshold) we included orange light (595 nm) correlating to the Chrimson excitation maximum. In the first set of experiments, we performed continuous photostimulation (5–60 s, irradiance 1–2 mW/mm^2^). Sharp microelectrode AP recordings showed that under baseline conditions, maximum negative diastolic potential in BiPOLES EHTs was less negative than in unedited control EHTs (−50 mV; Fig. 3A). Photostimulation with red (Chrimson activation) and blue (GtACR2 activation) light shifted the membrane potential to less negative potentials (with blue light to −24 mV; with red light to −14 mV) and resulted in a depolarization block (Fig. 3A–C). We then went on and performed continuous photostimulation while measuring contractility. In this setup, LEDs with much lower irradiance had to be used (maximal light intensity 0.013 mW/mm^2^ for blue and 0.010 mW/mm^2^ for red light). In accordance with the AP data, contractility measurements revealed that continuous photostimulation (that had no effect on control EHTs, Supplemental Figure 2) immediately stopped contractions in BiPOLES EHTs (Fig. 3D). Blue light photostimulation required only low light intensity. 18/46 EHTs stopped with 0.005 mW/mm^2^ and 40/46 EHTs stopped with higher light intensity (0.013 mW/mm^2^) which was the highest energy; the implemented LED could provide in the set-up for automated contractility measurements. Silencing BiPOLES EHTs was not possible while simultaneously measuring contractility with red light (maximal irradiance 0.010 mW/mm^2^). We therefore used a different setup and photostimulated EHTs with higher irradiance and recorded the time to the inhibition of contractility. In this setup, blue light inhibited contractility in 100% of the EHTs (Fig. 3E). Higher irradiance was required to stop EHT contractility with red light than with blue light (Fig. 3E). Yet, reaction to orange light (595 nm which matches the Chrimson excitation maximum) was similar as to blue light (Supplemental Figure 3A).

**Fig. 4:**
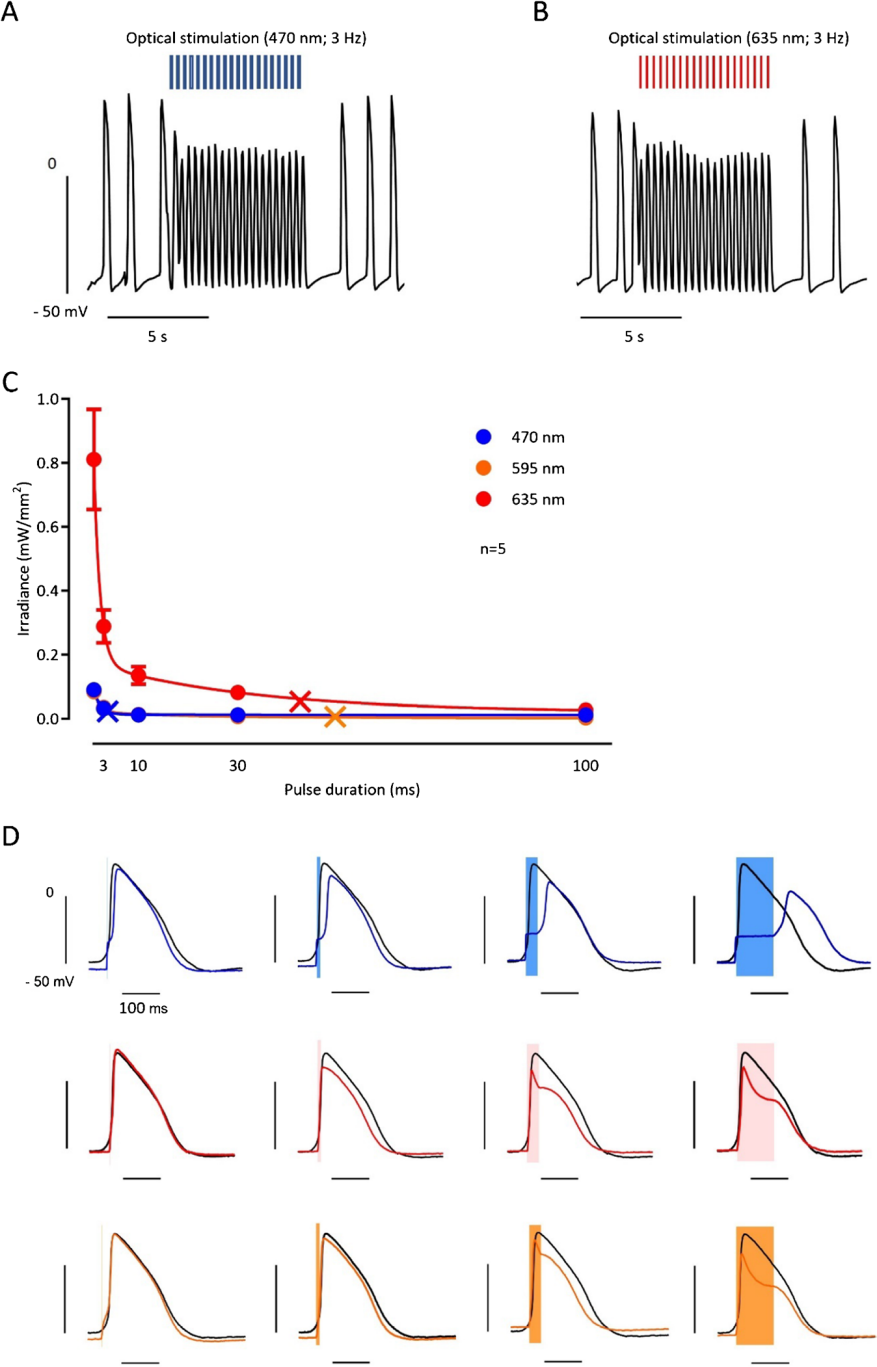

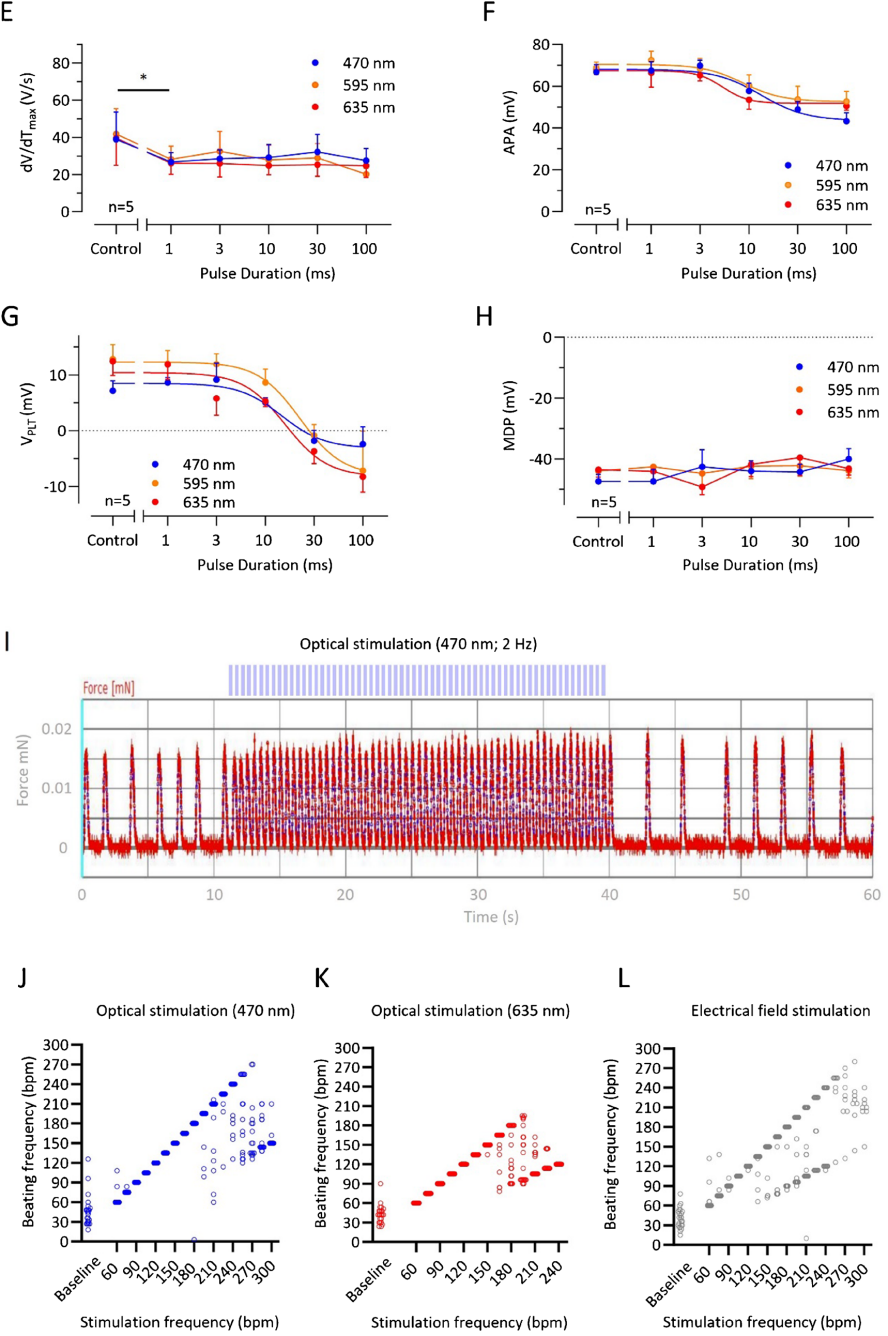
Pulsed stimulation of BiPOLES EHTs. **A**, **B** AP measurements with a sharp microelectrode in BiPOLES EHTs during baseline measurement and optical pacing with 3 Hz with **A** blue and **B** red light (irradiance 1–2 mW/mm^2^). **C** Strength–light pulse duration relationship in BIPOLES EHTs. Mean values ± SEM of threshold light activities to elicit an AP for different pulse duration of light pulses with a wavelength of 470, 595, and 635 nm. *n* = 5 BIPOLES EHTs. Monoexponential curves were fitted to data points to estimate rheobase and chronaxie (indicated by crosses). A nonlinear regression function was fitted to the data points. **D** Original traces of single AP in a BIPOLES EHT exposed to light pulse of increasing duration. A spontaneous action potential recorded in the same BIPOLES EHT is given in black for comparison (same control for all pulse durations). Pulse duration is indicated by the shaded area. **E**–**H** Mean values ± SEM for **E** maximal upstroke velocity (dV/dTmax), **F** AP amplitude (APA), **G** plateau voltage (V_PLT_), and **H** maximum diastolic potential (MDP) in five BIPOLES EHT for different pulse durations. Control indicates values obtained from spontaneous AP. Sigmoidal curves could be fitted to APA and V_PLT_ data points. *n* = number of BiPOLES EHTs. **I** Original contraction recording of a BiPOLES EHT during initiation of pulsed photostimulation (2 Hz). **J** Analysis of optical pacing capability with blue light (irradiance 0.013 mW/mm^2^, pulse duration 40 ms). **K** Analysis of optical pacing capability with red light (irradiance 0.010 mW/mm^2^, pulse duration 40 ms). **L** Analysis of optical pacing capability with electric field stimulation. One EHT represents one data point in J–L. *n* = 7–8 EHTs per batch/*n* = 3 batches

In a second set of experiments, we performed pulsed photostimulation. Three batches of EHTs (*n* = 7–8 EHTs per batch/*n* = 3 batches) were used. BiPOLES EHTs could be optically paced with pulsed photostimulation (Fig. 4A and B). To investigate the strength–duration relationship for the three different wavelengths, we measured threshold intensities at different pulse duration (Fig. 4C and Supplemental Figure 3B). For this purpose, we lowered the intensity every tenth beat until stimulation failed to elicit an AP (Supplemental Figure 3B). While resulting strength–duration curves for 595 and 635 nm light showed the expected shape with the typical flat tailing off in case of long pulse duration, the curve for 470 nm was much steeper (*p* < 0.05, *F*-test). Accordingly, chronaxie values were much lower: 4.9 ± 0.2 (vs. 54.7 ± 3.6 for 595 nm and 44.7 ± 9.9 for 635 nm, *p* < 0.05, *t* test; Fig. 4C and Supplemental Figure 3B). Having a closer look at AP induction, ETHs stimulated with longer pulses revealed a completely different mode of AP generation (Fig. 4D). While with lights of 595 nm and 635 nm wavelength, the AP initiated within the light pulse; in case of 470 nm, the AP started after light pulses have been terminated (Fig. 4D).

DV/dt_max_ was slightly but significantly lower with all three wavelengths at 1 ms pulse duration. No further depression of dV/dt_max_ occurred with longer pulse durations (Fig. 4E). AP amplitude (APA) and plateau voltage (V P_LT_) were depressed with pulse durations longer than 10 ms by light pulses of 595 and 635 but also with 470 nm (Fig. 4F and G). Maximal diastolic potential (MDP) was not affected even with longer pulse durations, indicating that channel activation has not impaired final repolarization (Fig. 4H).

To assess the impact of photostimulation on contractility, we optimized the pacing protocol for the video-optical measurement setup (Supplemental Figure 3C and D). Eventually, the light pulse duration was set at 40 ms with maximum irradiance (0.013 mW/mm^2^ for blue and 0.010 mW/mm^2^ for red light). We then performed experiments in three EHT batches. In two batches, all EHTs followed pacing with the blue light, up to a frequency of 210 bpm. Some EHTs even followed a maximal stimulation frequency up to 240 bpm with a 1:1 capture (maximal stimulation frequency with 1:1 capture was 270 bpm). However, most EHTs either showed a 2:1 or irregular capture with stimulation frequencies >210 bpm. The third EHT batch only followed optical pacing up to 165 bpm (Fig 4J and K). Similarly, EHTs could be optically paced with red light (635 nm; Fig. 4K and Supplemental Figure 4), where inter-EHT variability was again present. All BiPOLES EHTs followed pacing with the red light up to 150 bpm. EHTs from two batches showed an irregular capture with higher frequencies but EHTs from the third batch could be paced up to 195 bpm. Optogenetic stimulation capacity was compared to classical electrical field stimulation. To this end, BiPOLES EHTs were electrically paced at increasing frequencies (2.5 V, 4 ms). With electrical field stimulation, one BiPOLES EHT batch followed electrical pacing up to a frequency of 255 bpm, whereas the two other batches dropped out earlier (Fig. 4L). Values for individual batches can be found in Supplemental Figure 5A–C.

### Evaluation of all-trans-retinal supplementation

**Fig. 5:**
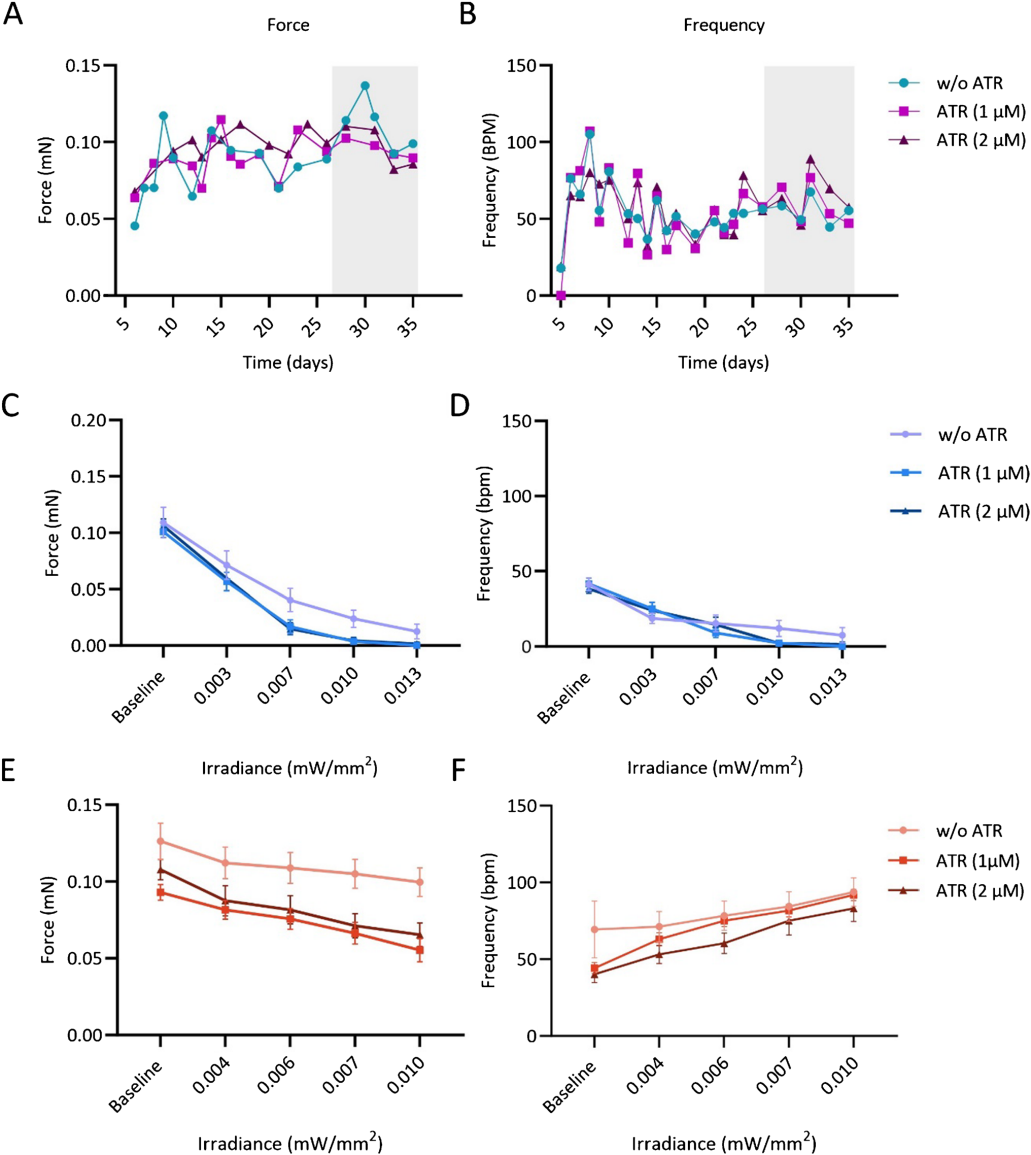
Evaluation of all-trans-retinal supplementation on BiPOLES EHT photosensitivity. **A** Force and **B** frequency measurements under ATR supplementation. ATR was added at day 27 of culture after an initial maturation phase of 4 weeks. **C** Force and **D** frequency measurement with increasing blue light intensity (irradiance in mW/mm^2^). **E** Force and **F** frequency measurement with increasing red light intensity (irradiance in mW/mm^2^). **C**, **D** Baseline represents low background illumination with green light *E* = 0.001 mW/mm^2^. **F** Depicted are mean data from 2 EHT batches (*n* = 8 EHTs per batch) ± SEM. ATR, all-trans-retinal

Finally, we investigated whether supplementation of all-trans-retinal (ATR) which has previously been shown to enhance photosensitivity of channelrhodopsin 2 in virally transduced neonatal rat cardiomyocytes [52] also enhances photosensitivity in BiPOLES EHTs. ATR supplementation (1 μM and 2 μM) had no effect on EHT contractility and frequency (incubation period 7 days), indicating that it had no toxic effect in the applied concentrations (Fig. 5A and B). We then performed continuous photostimulation with increasing light intensity while measuring frequency and contractile force. For this, light was continuously applied for 10 s, and contractility and frequency were measured (*n* = 16 EHTs from 2 batches). Continuous light application resulted in a decrease in force for either blue (470 nm, maximal irradiance 0.013 mW/mm^2^) or red light (635 nm, maximal irradiance 0.010 mW/mm^2^). Beating frequency increased during continuous light application with red light. For blue light, the beating frequency decreased on average which was mainly caused by a beating arrest of some EHTs already under low-intensity blue light illumination (data for individual EHTs is shown in Supplemental Figure 6).

## Discussion

Modulation of cardiomyocyte physiology with light offers a plethora of application possibilities. In this study, we took advantage of BiPOLES, an optogenetic tool that enables spectrally distinct activation of cation or anion conductances in the same stem cell–derived EHTs. There were three major findings: (i) activation, i.e., pulsed stimulation with blue (470 nm, anion conductance by GtACR2 activation) as well as with red light (635 nm, cation conductance by Chrimson activation) could be used to optically pace EHTs; (ii) sustained illumination, either with red or with blue light reversibly silenced cardiac contractility within seconds; (iii) lower irradiance was required for activation with blue than with red light but activation irradiance for orange light (that matches the Chrimson excitation maximum) was similar to blue light.

In cardiovascular science, optogenetic tools are attractive for both activating but also silencing cardiac activity. On the activation side (i.e., pacing), they are attractive because electrical field stimulation is limited by the generation of toxic products (e.g., generation of chlorine, hydroxyl radicals, and hypochlorous acid) [19] in vitro and technically challenging in small animal models [18]. Stimulation has been used to model tachyarrhythmias [27, 29], study cell–cell interactions [21], control heart rate [20], and, in more translational approaches, evaluate optical pacemaking [35]. For this purpose, mainly cation channels have been used. In accordance, pulsed Chrimson activation was able to stimulate BiPOLES EHTs. To date, only one study reported the activation of anion channels to excite cardiomyocytes [24]. Our results demonstrating optical stimulation by activation of GtACR2 are in line with this study. At first sight, this finding may seem surprising, as activation of anion channels has been used repeatedly to inhibit neuronal firing. Yet, the maximal diastolic potential in stem cell–derived engineered heart tissue, similar as in human left-ventricular myocardium, lies between −70 and −80 mV [28]. The reversal potential for chloride in cardiomyocytes is ~−30 mV [25]. Transient (pulsed) activation of a chloride conducting channel therefore depolarizes the membrane potential in cardiomyocytes, triggering an AP. Yet, even though red and orange (Chrimson activation) and blue light (GtACR2 activation) elicited AP in BiPOLES, experiments with longer light pulses revealed that the mode of AP generation differed fundamentally. Excitation by light pulses at 595 and 635 nm was mediated by a rapid drop (less negative) in diastolic potential that can be strong enough to reach the activation threshold for calcium currents. *E*_rev_ for the cation current that dominates membrane potential during photostimulation is sufficiently above the activation threshold for *I*_Ca,L_ in hiPSC-CM allowing almost full activation of large peak *I*_Ca,L_ currents as indicated by only slightly depressed *V*_max_. Thus, longer pulses at 595 and 635 nm cannot impact *V*_max_ further. However, longer pulses can affect the plateau phase (beginning with peak of AP, as indicated by values for APA) when *I*_Ca,L_ currents declined slowly, thereby exaggerating effects of the stable light-induced conductivity on membrane potential. The situation was at variance with light of 470 nm, where AP did not start before light cessation. Here, the *E*_rev_ lies at ~−35 mV. In principle, *I*_Ca,T_ (that is present in hiPSC-CM) [45] should get activated at that membrane voltage. However, we would expect that the slightly depolarized MDP in BIPOLES EHT will keep *I*_Ca,T_ steady-state inactivated. We can only speculate on the mode of AP generation in case of 470 nm. Obviously, a persistent depolarizing current must be activated during the light application. Small enough to not overcome the *E*_rev_ at −35 mV as long as the artificial conductivity persists, but strong enough to putatively activate I_Ca,L_ when the artificial conductivity (i.e., light pulse) abruptly ends. Computer simulations may help to better understand the mechanisms of “post”-light stimulation by optogenetics.

On the inactivation side, prolonged photostimulation can be used to silence cardiomyocytes. This strategy has been applied for optical defibrillation [6, 9, 14]. We have recently used this strategy to specifically inhibit transplanted cardiomyocytes in the injured heart to dissect out the mechanism by which engrafted cardiomyocytes support left-ventricular function [44]. Inhibiting cardiomyocyte contractility can also be of interest for analyzing the effect of cardiac work on maturation in pluripotent stem cell–derived myocytes but also to replace pharmacological approaches when studying the (molecular) consequences of unloading [43]. In BiPOLES EHTs, prolonged photostimulation with blue as well as with red light stopped contractility, which can be explained by a shift of the maximal diastolic membrane potential to less negative values, thereby inactivating sodium channels and locking the cardiomyocytes in a depolarized state (and inhibiting excitation by shunting due to the large conductance). Alternatively, silencing cardiomyocytes could be achieved by the activation of proton pumps [14] or via the opening of potassium channels as recently described with the optogenetic tool WiChR [46]. Combining a potassium channel and a chloride conducting anion channel or a channelrhodopsin could therefore be better suited to generate a tool for bidirectional modulation of cardiomyocyte physiology.

The light intensity that was required to stimulate but also to silence BiPOLES EHTs was lower for GtACR2 (blue light) than for Chrimson (red light). However, when using orange light that better fits the excitation maximum for Chrimson, there was no difference in required light intensity between GtACR2 and Chrimson. This is of particular interest because the stoichiometry between GtACR2 and Chrimson in BiPOLES is fixed to 1:1. This finding indicates that anion channels can serve as an alternative to classically used channelrhodopsin for optical pacing as well as for defibrillation purposes but does not provide evidence for one strategy being superior to the other. Overall, the light intensity required for stimulation and inhibition was similar to other studies with BiPOLES [47] but also studies with other optogenetic tools [5, 53]. Yet, one limitation of our work is the low light intensity that can be applied in the video-optical measurement system. For prospective future in vivo application of anion channels, a red-shifted anion channel with better tissue penetrance of light might be favorable. ATR supplementation was previously described to enhance light sensitivity and viability [52]. Even though there was a tendency to increased light sensitivity, no major impact of ATR supplementation was observed on BiPOLES EHTs.

BiPOLES EHTs remained lower in force and had more variable frequencies than control EHTs. Force measurements required some background illumination with green light (525 nm, 0.001 mW/mm^2^) for the figure recognition software. The fact that contractility was not higher when analyzed with infrared background illumination (which does not activate BiPOLES) argues against an effect of green backlight illumination. Yet, the analysis was technically more difficult with low-intensity green light illumination which might partially explain the greater variability in the physiological parameters of BiPOLES EHTs. Histological analysis showed no difference in cardiomyocyte structure and orientation between BiPOLES EHTs and EHTs derived from unedited cardiomyocytes, indicating that cardiomyocyte maturation was not affected by the BiPOLES expression and therefore cannot explain the functional differences. Alternatively, transgene expression could exert off-target effects. We had previously observed similar effects on cardiac physiology (lower contractility and a more positive diastolic membrane potential) with the optogenetic tool iChloc [44] but also when overexpressing chemogenetic tools (i.e., pharmacological selective actuator modules, PSAMs) [44].

In summary, we demonstrate that engineered cardiac constructs can be optically silenced as well as optically paced by the activation of anion channels as well as cation channels with high frequency providing a useful in vitro platform to study cardiovascular physiology and pathophysiology.

### Supplementary information

ESM 1 (PDF 542 kb)

## Data Availability

All the data supporting the findings from this study are available within the article and its supplementary information are available from the corresponding author upon request.
