# Supplementary material for: Modulating cardiac physiology in engineered heart tissue with the bidirectional optogenetic tool BiPOLES
Source: Pflugers Arch. 2023 Oct 21;475(12):1463–77. doi: 10.1007/s00424-023-02869-x (PMC10730631; doi:10.1007/s00424-023-02869-x)
Supplement: Supplementary file 1 — (PDF 542 kb) [file 424_2023_2869_MOESM1_ESM.pdf]

Supplemental Figures

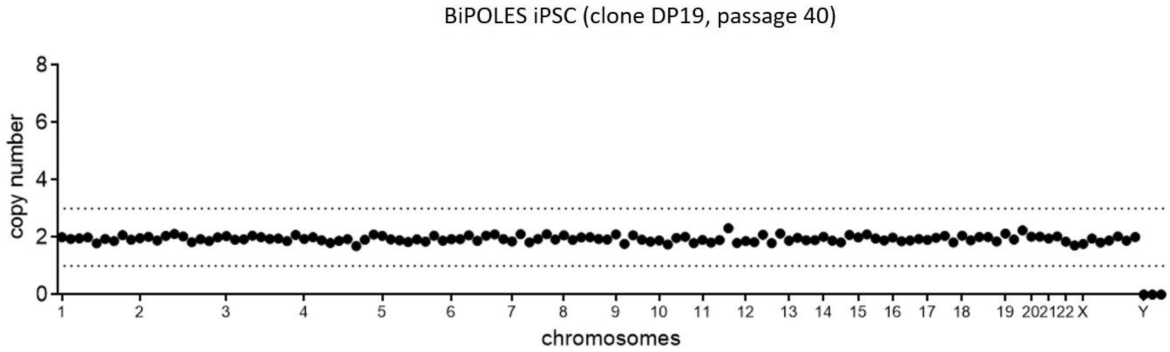

Supplemental Figure 1: Nanostring based karyotyping analysis of BiPOLES iPSC. Note that all probes display regular copy number (between dotted lines), arguing against chromosome structural aberrances.

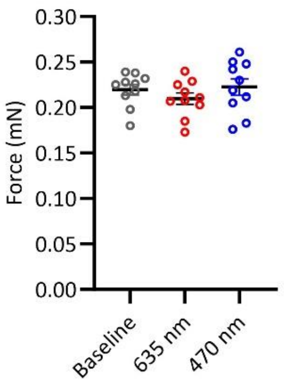

Supplemental Figure 2: Photostimulation of EHTs derived from unedited cardiomyocytes. Irradiance: 0.013 mW/mm^2 for 470 nm and 0.010 mW/mm^2 for 635 nm.

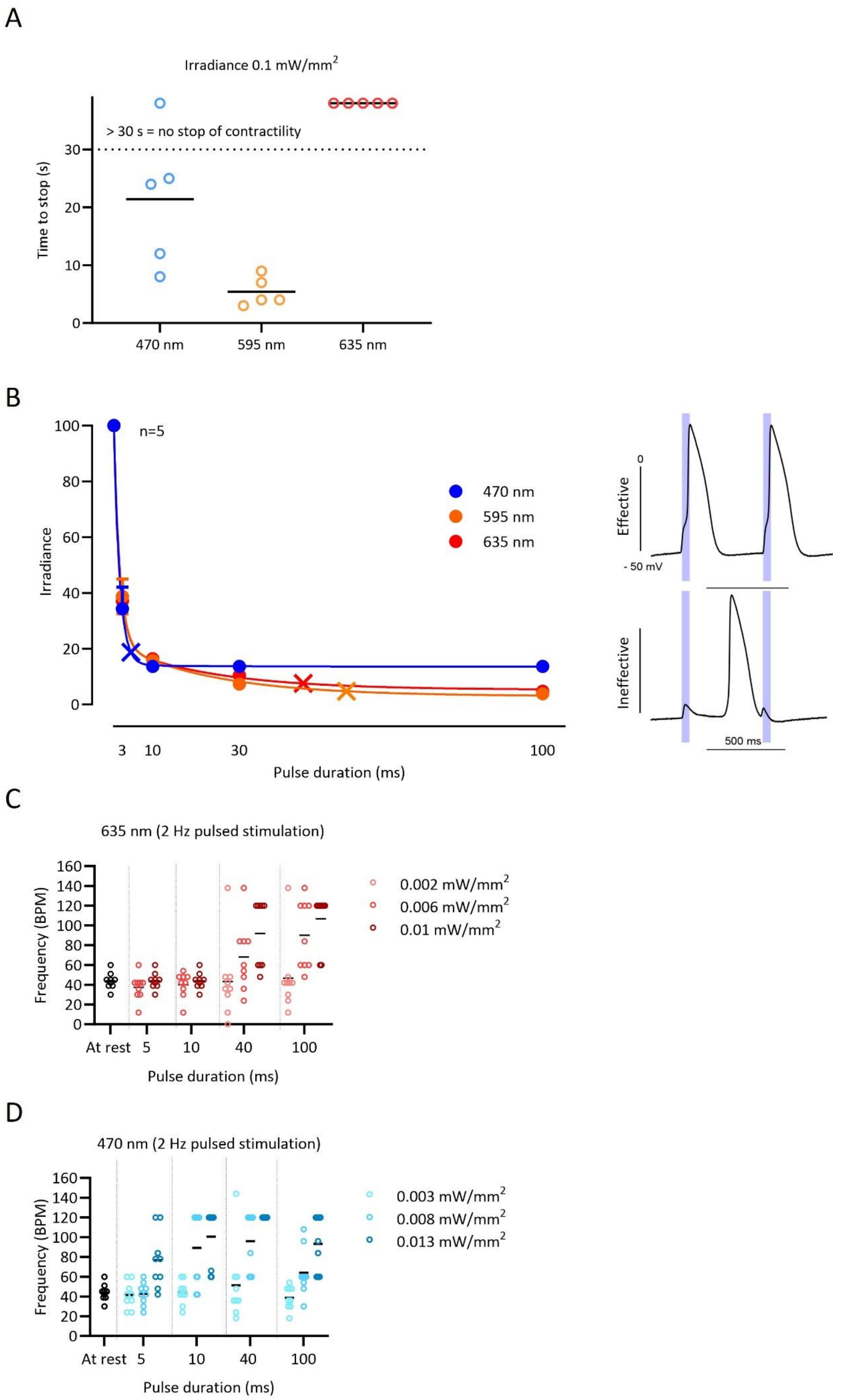
A
Irradiance 0.1 mW/mm2
> 30 s = no stop of contractility
Time to stop (s)
0
10
20
30
470 nm
595 nm
635 nm
B
n=5
Irradiance
0
20
40
60
80
100
470 nm
595 nm
635 nm
3
10
30
100
Pulse duration (ms)
Effective
0
- 50 mV
Ineffective
500 ms
C
635 nm (2 Hz pulsed stimulation)
Frequency (BPM)
0
20
40
60
80
100
120
140
160
At rest
5
10
40
100
Pulse duration (ms)
0.002 mW/mm2
0.006 mW/mm2
0.01 mW/mm2
D
470 nm (2 Hz pulsed stimulation)
Frequency (BPM)
0
20
40
60
80
100
120
140
160
At rest
5
10
40
100
Pulse duration (ms)
0.003 mW/mm2
0.008 mW/mm2
0.013 mW/mm2

Supplemental Figure 3: Pulsed photostimulation of BiPOLES EHTs. A) Time to stop of EHT contractility with blue (470 nm), orange (590 nm) and red (635 nm) light at 0.010 mW/mm^2 irradiance. **B**) Normalized strength-duration relationship in BiPOLES EHT. Summary of results: Mean values ± SEM of threshold light activities to elicit an AP for different pulse duration of light pulses with a wavelength of 470, 595 and 635 nm. Monoexponential curves were fitted to data points to estimate rheobase and chronaxie (indicated by crosses). Right: Original recordings of membrane potential recorded in BiPOLES EHT exposed to 10 ms long pulses at 470 nm wavelength at an intensity sufficient (top) and insufficient (below) to elicit an AP. Please note small deviations of membrane potential, not reaching the threshold for a self-generating upstroke. **C and D**) Optimization of the pacing strategy for the video-optical recording system. Photostimulation was performed with increasing pulse and light durations for (C) red (635 nm) and (D) blue (470 nm) at 2 Hz.

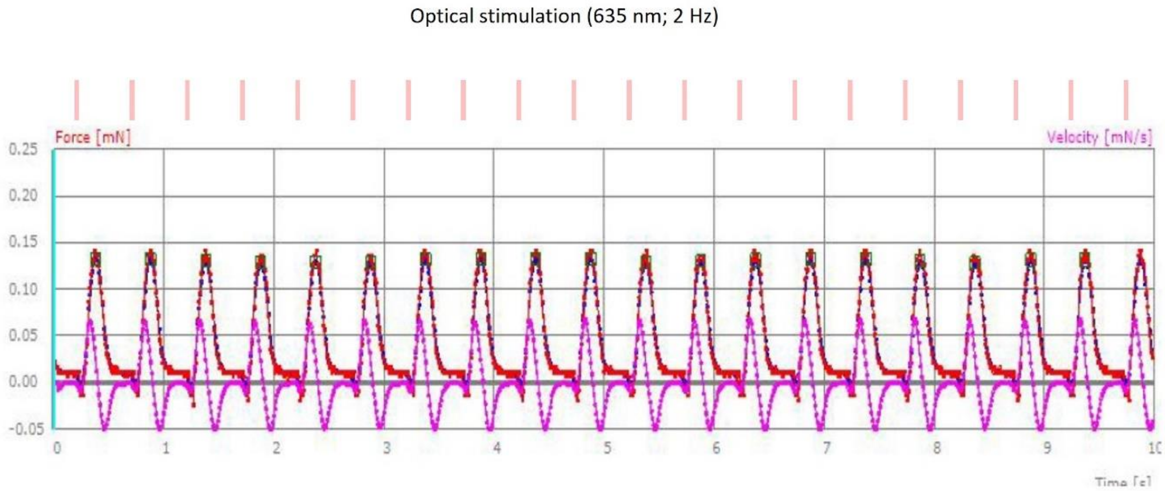

Supplemental Figure 4: Original recording of a BiPOLES EHT under pulsed photostimulation with red light. Irradiance 0.010 mW/mm^2.

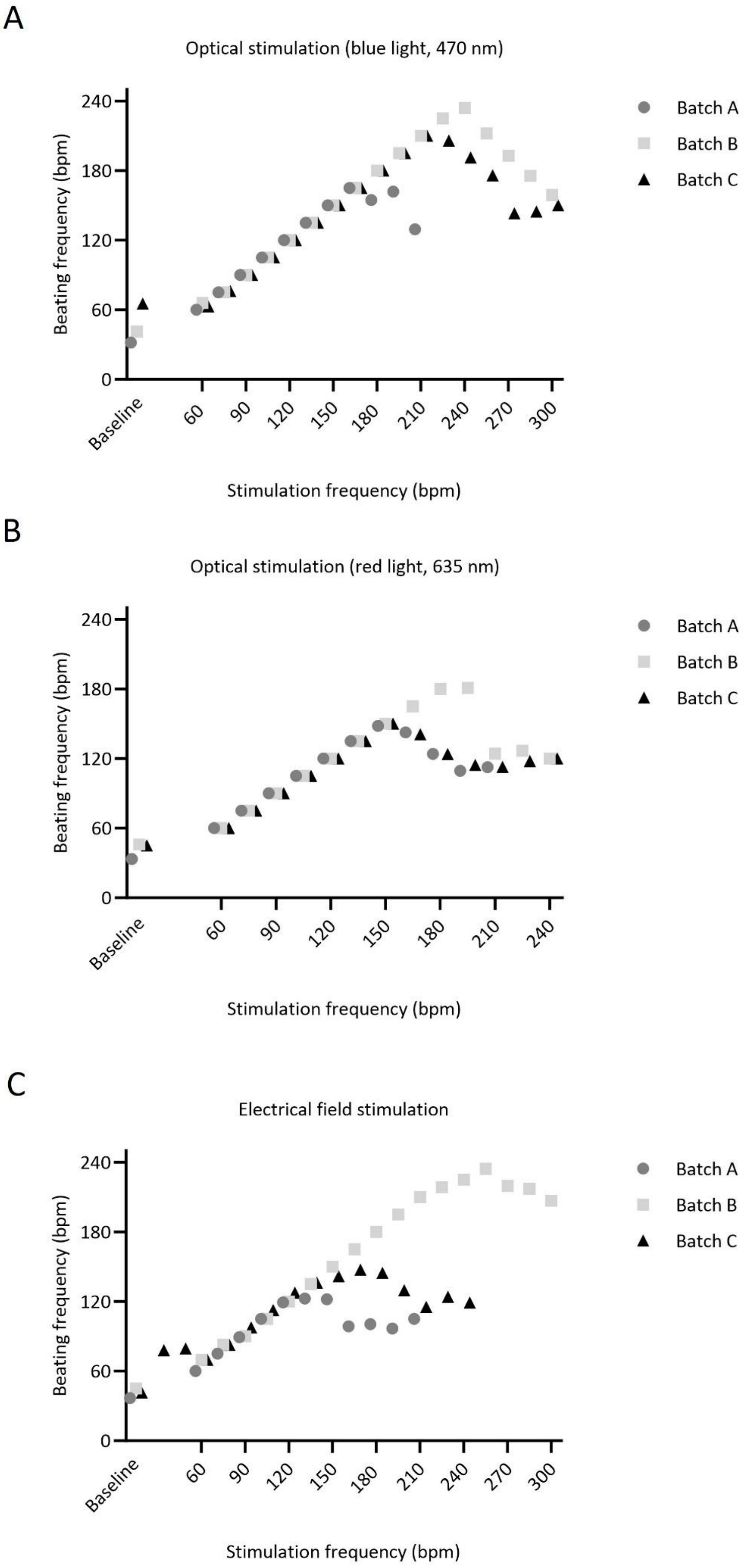
A
Optical stimulation (blue light, 470 nm)
Beating frequency (bpm)
240
180
120
60
0
Baseline
60
90
120
150
180
210
240
270
300
Stimulation frequency (bpm)
Batch A
Batch B
Batch C
B
Optical stimulation (red light, 635 nm)
Beating frequency (bpm)
240
180
120
60
0
Baseline
60
90
120
150
180
210
240
Stimulation frequency (bpm)
Batch A
Batch B
Batch C
C
Electrical field stimulation
Beating frequency (bpm)
240
180
120
60
0
Baseline
60
90
120
150
180
210
240
270
300
Stimulation frequency (bpm)
Batch A
Batch B
Batch C

Supplemental Figure 5: Pulsed photostimulation of BiPOLES EHTs three individual EHT batches are shown respectively.

A

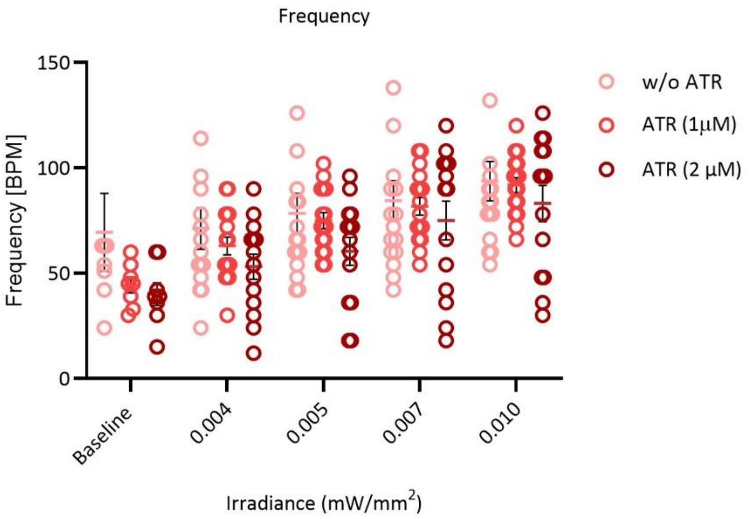

B

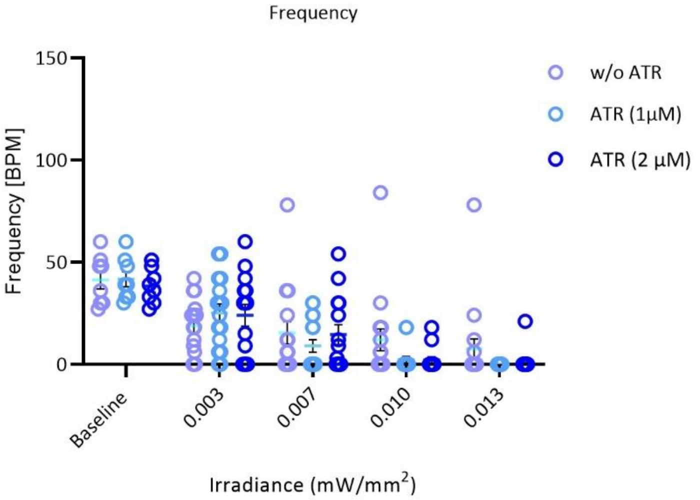

Supplemental Figure 6: Evaluation of all-trans retinal supplementation on BiPOLES EHT photosensitivity. Each data point represents one EHT.
